# Nosocomial infections in sickle cell anemia patients: Prevention through multi-disciplinary approach: A review

**DOI:** 10.1097/MD.0000000000036462

**Published:** 2023-12-01

**Authors:** Emmanuel Ifeanyi Obeagu, Getrude Uzoma Obeagu, Callistus Akinleye Akinleye, Matthew Chibunna Igwe

**Affiliations:** a Department of Medical Laboratory Science, Kampala International University, Uganda; b School of Nursing Science, Kampala International University, Uganda; c Department of Community Medicine, Osun State University, Osogbo, Nigeria; d Department of Public Health, Kampala International University, Uganda.

**Keywords:** anemia, infection, nosocomial infection, sickle cell anemia

## Abstract

Sickle cell anemia (SCA), a hereditary blood disorder characterized by the presence of abnormal hemoglobin, poses a unique set of challenges for both patients and healthcare providers. One of the most pressing issues in the care of these individuals is the persistent threat of nosocomial infections, which are infections acquired during hospitalization. This abstract provides a concise overview of the ongoing challenge of nosocomial infections in SCA patients, highlighting the factors contributing to their vulnerability and the preventive measures in place. SCA patients face increased susceptibility to nosocomial infections due to their compromised immune systems, frequent hospitalizations, prolonged stays, and the need for invasive medical interventions. The emergence of multidrug-resistant pathogens further complicates the management of these infections. To address this challenge, healthcare facilities have implemented infection control protocols, vaccination strategies, and antimicrobial stewardship, emphasizing the importance of patient education. Recognizing the gravity of this issue and adopting comprehensive preventive measures is crucial to improving the quality of care and patient outcomes in this vulnerable population. Further research and ongoing efforts are essential to reducing the burden of nosocomial infections in SCA patients and enhancing their overall healthcare experience.

## 1. Introduction

Sickle cell anemia (SCA), a hereditary blood disorder characterized by the presence of abnormal hemoglobin, presents an enduring and complex challenge in healthcare. Among the myriad complications faced by individuals with this condition, one of the most pressing and persistent threats is the occurrence of nosocomial infections. These infections, which are acquired during the course of healthcare delivery, pose a critical concern for patients and healthcare providers alike, as they significantly impact patient outcomes, healthcare costs, and overall patient safety.^[1–4]^

SCA patients experience a unique set of vulnerabilities that predispose them to nosocomial infections. These vulnerabilities stem from both the nature of the disease and the medical interventions required to manage its symptoms. Individuals afflicted with SCA suffer from a weakened immune system, frequent and often prolonged hospitalizations, and a high likelihood of requiring invasive medical procedures. These factors collectively heighten their susceptibility to infections acquired within healthcare facilities, further compounded by the emergence of multidrug-resistant pathogens.^[5–8]^

This article delves into the ongoing challenge of nosocomial infections in SCA patients, exploring the contributing factors and highlighting the preventive measures and interventions that healthcare providers have implemented to mitigate this pressing issue. Recognizing the gravity of this challenge and the imperative to address it comprehensively is central to improving the quality of care and patient outcomes in this vulnerable patient population.^[9]^ Furthermore, ongoing research and vigilance are essential to reducing the burden of nosocomial infections and enhancing the overall healthcare experience for individuals battling SCA.

## 2. Nosocomial infections: a continuing threat

Nosocomial infections, often referred to as hospital-acquired infections (***HAIs) or healthcare-associated infections (HAIs), are a persistent and alarming challenge within healthcare systems worldwide. These infections encompass a diverse range of bacterial, viral, and fungal pathogens acquired during the course of healthcare delivery within hospitals, clinics, long-term care facilities, and other healthcare settings. Despite significant advancements in medical science and infection control measures, nosocomial infections continue to pose a substantial threat to patient safety, medical outcomes, and healthcare costs.^[10,11]^

Several key factors contribute to the ongoing challenge of nosocomial infections:^[10,11]^

1. Weakened immune defenses: Many patients within healthcare facilities are already coping with serious medical conditions, which often weaken their immune systems. This compromised immunity makes them more susceptible to infections, even from microorganisms that might be harmless to those with robust immune defenses.
2. Invasive medical interventions: A significant number of patients require invasive procedures such as surgery, catheterization, and mechanical ventilation. These interventions, while essential for treatment, create opportunities for pathogens to enter the body and cause infections.
3. Prolonged hospital stays: Longer hospital stays are often necessary for patients with complex or severe medical conditions. The duration of exposure to healthcare environments increases the risk of encountering infectious agents, especially in crowded or high-traffic areas.
4. Antibiotic resistance: The emergence of antibiotic-resistant strains of bacteria, often called “superbugs,” has complicated the treatment of nosocomial infections. These pathogens do not respond to standard antibiotic treatments, leading to increased morbidity and mortality rates.
5. Inadequate infection control practices: Insufficient adherence to infection control practices, such as hand hygiene, proper sterilization of equipment, and environmental cleaning, can contribute to the persistence of nosocomial infections.

Addressing the continuing threat of nosocomial infections requires a multifaceted approach:^[12,13]^

1. Preventive strategies: Rigorous implementation of preventive measures, including hand hygiene, proper sterilization of equipment, and the use of personal protective equipment, is essential to reduce the risk of transmission.
2. Vaccination: Encouraging vaccination among healthcare workers and patients can mitigate the risk of specific infections, such as influenza and hepatitis.
3. Antimicrobial stewardship: Promoting responsible antibiotic use helps combat the emergence of drug-resistant pathogens and ensures that patients receive appropriate treatment.
4. Patient education: Educating patients about infection prevention, hygiene, and the importance of following medical instructions can empower them to actively participate in reducing infection risk.
5. Surveillance and research: Continuous monitoring and research efforts are necessary to identify emerging pathogens, understand their transmission patterns, and develop improved infection control strategies.

Nosocomial infections remain a substantial challenge within healthcare, endangering patient well-being and straining healthcare resources. Addressing this ongoing threat requires the collective efforts of healthcare providers, policymakers, researchers, and patients to implement and adhere to robust infection control practices and stay vigilant in the face of evolving infectious agents. Only through a comprehensive and sustained approach can we hope to mitigate the persistent menace of nosocomial infections and provide safer, more effective healthcare for all.^[14,15]^

***Top of Form

Bottom of Form

## 3. Factors contributing to the challenge of nosocomial infections in SCA

SCA is a hereditary blood disorder characterized by the presence of abnormal hemoglobin, resulting in the formation of misshapen red blood cells. While this condition is associated with a range of complications, one of the most daunting challenges confronting both patients and healthcare providers is the persistent threat of nosocomial infections. The unique vulnerabilities of SCA patients make them particularly prone to these hospital-acquired infections. Several interrelated factors contribute to this ongoing challenge:^[16–19]^

1. Frequent hospitalizations: SCA patients experience recurrent and often severe symptoms, including vaso-occlusive crises, acute chest syndrome, and severe anemia. As a result, they require frequent hospitalizations for pain management, blood transfusions, and treatment of complications. Each hospitalization presents an opportunity for the acquisition of nosocomial infections.
2. Compromised immune function: SCA is characterized by a weakened immune system. The chronic nature of the disease and the frequent use of immunosuppressive medications, such as hydroxyurea, can further suppress the immune response. This immunodeficiency leaves patients less equipped to defend against infectious agents encountered in healthcare settings.
3. Invasive procedures: SCA patients often require a range of invasive medical interventions, including central venous catheter placements, blood transfusions, and surgical procedures. These procedures, while essential for managing their condition, increase the likelihood of exposure to potential sources of infection.
4. Prolonged hospital stays: SCA patients often experience extended hospital stays, as they may encounter complications, require close monitoring, or have protracted recovery periods. The longer the hospitalization, the greater the duration of exposure to healthcare environments and the increased risk of encountering infectious agents.
5. Multidrug-resistant pathogens: The emergence of multidrug-resistant pathogens poses a significant challenge in the treatment of nosocomial infections in SCA patients. These infections may be more challenging to manage, leading to increased morbidity and mortality rates.
6. Overlapping conditions: SCA patients may also have other concurrent health conditions, such as chronic organ damage or respiratory problems, which further compromise their health and increase their susceptibility to infections.

Addressing the multifaceted challenge of nosocomial infections in SCA patients requires a comprehensive approach:^[19]^

1. Infection control protocols: Healthcare facilities must implement and strictly adhere to infection control protocols, emphasizing hand hygiene, environmental cleaning, and the proper sterilization of equipment.
2. Vaccination: Ensuring that SCA patients are up-to-date on recommended vaccinations, such as those for influenza and pneumonia, can help reduce their risk of these infections.
3. Central line-associated bloodstream infection (CLABSI) prevention: Proper maintenance of central venous catheters and adherence to CLABSI prevention bundles can significantly reduce the risk of these infections.
4. Antimicrobial stewardship: Healthcare providers should judiciously prescribe antibiotics to prevent the emergence of antibiotic-resistant strains and to ensure appropriate treatment.
5. Patient education: Empowering SCA patients with knowledge about infection prevention and the importance of proactive self-care can play a pivotal role in reducing infection risk.

The factors contributing to the challenge of nosocomial infections in SCA patients are diverse and complex. Healthcare providers, researchers, and policymakers must work collaboratively to develop and implement strategies that address these factors and reduce the risk of nosocomial infections. By doing so, we can improve the quality of care and overall healthcare outcomes for this vulnerable patient population.^[20,21]^

## 4. Preventive measures and interventions for nosocomial infections in SCA

The management of nosocomial infections in SCA patients is a pressing issue that demands a multifaceted approach. Given the unique vulnerabilities of these individuals, the implementation of robust preventive measures and interventions is essential to reduce the risk of nosocomial infections. Below, we discuss various strategies and actions aimed at addressing this ongoing challenge:^[22,23]^

### 4.1. Infection control protocols

#### **Hand hygiene:

Encouraging strict hand hygiene compliance among healthcare providers is a fundamental preventive measure. Proper and frequent handwashing with soap and water or the use of alcohol-based hand sanitizers significantly reduces the transmission of infectious agents.

#### *Environmental cleaning*:

Regular and thorough cleaning of patient care areas, surfaces, and equipment is crucial to minimize the reservoir of potential pathogens. Adequate disinfection practices should be upheld.

#### *Isolation precautions*:

Implementing isolation precautions for infected or colonized patients can prevent the spread of infections to others. This is particularly relevant for multidrug-resistant pathogens.

### 4.2. Vaccination

Ensuring that SCA patients are up-to-date on recommended vaccinations is vital. Vaccines for influenza, pneumonia, and other preventable diseases should be administered to reduce the risk of these infections.

### 4.3. CLABSI prevention

Implementing CLABSI prevention bundles can be highly effective. These bundles include strict aseptic techniques during catheter insertion and maintenance, proper dressing changes, and minimizing the duration of catheter use.

### 4.4. Antimicrobial stewardship

Healthcare providers must practice responsible antibiotic use to prevent the emergence of antibiotic-resistant strains of bacteria. Antibiotics should only be prescribed when necessary, and targeted therapies should be employed to ensure effective treatment.

### 4.5. Patient education

Empowering SCA patients with knowledge about infection prevention and self-care is crucial. Patients should be educated on the importance of reporting any signs or symptoms of infection promptly. They should also understand the necessity of adhering to their treatment plans and medication regimens.

### 4.6. Regular surveillance

Continuous monitoring of nosocomial infection rates in SCA patients is essential. Hospitals should establish surveillance programs to detect trends, identify risk factors, and respond to emerging infectious threats promptly.

### 4.7. Multi-disciplinary approach

Collaboration among healthcare providers, including hematologists, infectious disease specialists, nurses, and infection control teams, is essential. A multi-disciplinary approach can ensure that best practices are implemented throughout the patient care journey.

### 4.8. Innovative technologies

Utilizing advanced technologies, such as electronic health records and laboratory information systems, can aid in identifying high-risk patients and potential infection sources. These tools can also help with timely notification and intervention.

### 4.9. Research and development

Ongoing research is vital to better understand the unique risks and needs of SCA patients. This research can lead to the development of novel interventions, therapies, and infection control strategies tailored to this specific patient population.

Preventing nosocomial infections in SCA patients is an ongoing challenge in healthcare. By diligently implementing the preventive measures and interventions outlined above, healthcare providers can significantly reduce the risk of these infections and enhance the quality of care for this vulnerable patient group. Continued research and collaborative efforts are essential to address this ongoing challenge effectively.^[24]^Top of Form

Bottom of Form

Top of Form

Bottom of Form

## 5. Conclusion

Nosocomial infections in SCA patients constitute an enduring and complex challenge in healthcare. These individuals, already burdened by the relentless nature of their hereditary blood disorder, are at heightened risk of acquiring infections during their interactions with healthcare settings. The factors contributing to this ongoing challenge, including weakened immune function, frequent hospitalizations, invasive procedures, prolonged hospital stays, and the emergence of multidrug-resistant pathogens, make it a multifaceted concern that demands unwavering attention.

However, through a combination of rigorous preventive measures and targeted interventions, healthcare providers and policymakers have made significant strides in addressing this issue. Infection control protocols, vaccination programs, antimicrobial stewardship, patient education, and a collaborative, multi-disciplinary approach have all played essential roles in reducing the risk of nosocomial infections. These strategies not only protect patients but also benefit the healthcare system by reducing healthcare-associated infection rates and the associated financial burden.

Yet, despite these advances, vigilance and dedication to the cause remain paramount. The landscape of infectious diseases is ever-evolving, necessitating ongoing research and adaptability to address emerging threats. As we continue to learn more about the unique risks and needs of SCA patients, it is incumbent upon healthcare providers, researchers, and policymakers to adapt and innovate their approaches accordingly.

In conclusion, nosocomial infections in SCA patients will remain a challenge, but it is a challenge that can be met with the right measures in place. By staying committed to the best practices in infection prevention and continuously refining our understanding of the vulnerabilities of these patients, we can aspire to reduce the burden of nosocomial infections and provide better healthcare outcomes for this vulnerable patient population. This ongoing effort underscores the imperative of compassionate, patient-centered care and the enduring commitment of the healthcare community to patient safety and well-being.

## Author contributions

**Conceptualization:** Emmanuel Ifeanyi Obeagu.

**Methodology:** Emmanuel Ifeanyi Obeagu, Getrude Uzoma Obeagu.

**Supervision:** Emmanuel Ifeanyi Obeagu.

**Validation:** Emmanuel Ifeanyi Obeagu.

**Visualization:** Emmanuel Ifeanyi Obeagu.

**Writing – original draft:** Emmanuel Ifeanyi Obeagu, Getrude Uzoma Obeagu, Callistus Akinleye Akinleye, Matthew Chibunna Igwe.

**Writing – review & editing:** Emmanuel Ifeanyi Obeagu, Getrude Uzoma Obeagu, Callistus Akinleye Akinleye, Matthew Chibunna Igwe.

## References

[1] Obeagu EI Ochei KC Nwachukwu BN. Sickle cell anaemia: a review. Scholars J Appl Med Sci. 2015;3:2244–52.

[2] Obeagu EI. Erythropoeitin in sickle cell anaemia: a review. Int J Res Stud Med Health Sci. 2020;5:22–8.

[3] Obeagu EI. Sickle cell anaemia: haemolysis and anemia. Int J Curr Res ChemPharm Sci. 2018;5:20–1.

[4] Obeagu EI Muhimbura E Kagenderezo BP. An update on interferon gamma and C reactive proteins in sickle cell anaemia crisis. J Biomed Sci. 2022;11:84.

[5] Obeagu EI Bunu UO Obeagu GU. Antioxidants in the management of sickle cell anaemia: an area to be exploited for the wellbeing of the patients. Int Res Med Health Sci. 2023;6:12–7.

[6] Obeagu EI. An update on micro-RNA in sickle cell disease. Int J Adv Res Biol Sci. 2018;5:157–8.

[7] Obeagu EI Ogunnaya FU Obeagu GU. Sickle cell anaemia: a gestational enigma. Migration. 2023;17:18.

[8] Obeagu EI Dahir FS Francisca U. Hyperthyroidism in sickle cell anaemia. Int J Adv Res Biol Sci. 2023;10:81–9.

[9] World Health Organization. Noncommunicable Disease, Mental Health Cluster. Innovative Care for Chronic Conditions: Building Blocks for Action: Global Report. World Health Organization. 2002.

[10] Lemiech-Mirowska E Kiersnowska ZM Michałkiewicz M. Nosocomial infections as one of the most important problems of healthcare system. Ann Agric Environ Med. 2021;28. 10.26444/aaem/122629 34558254

[11] Breathnach AS. Nosocomial infections and infection control. Medicine (Baltimore). 2013;41:649–53.

[12] Martin-Loeches I Metersky M Kalil A. Strategies for implementation of a multidisciplinary approach to the treatment of nosocomial infections in critically ill patients. Expert Rev Anti Infect Ther. 2021;19:759–67. 33249874 10.1080/14787210.2021.1857730

[13] Stock NK Petráš P Melter O. Importance of multifaceted approaches in infection control: a practical experience from an outbreak investigation. PLoS One. 2016;11:e0157981. 27322433 10.1371/journal.pone.0157981 PMC4913898

[14] Allegranzi B Storr J Dziekan G. The first global patient safety challenge “clean care is safer care”: from launch to current progress and achievements. J Hosp Infect. 2007;65:115–23. 17540254 10.1016/S0195-6701(07)60027-9

[15] Haque M McKimm J Sartelli M. Strategies to prevent healthcare-associated infections: a narrative overview. Risk Manag Health Policy. 2020:1765–80. 10.2147/RMHP.S269315 PMC7532064 33061710

[16] Sadhu T Paned B Vera HK. Infection and potential challenge of childhood mortality in sickle cell disease: a comprehensive review of the literature from a global perspective. Thalassemia Rep. 2023;13:206–29.

[17] Mani CS. Acute pneumonia and its complications. Prince Pact Pediatric infectious Dis. 2018:238.

[18] Shabbat R Bell BP Schacht A. Emerging and reemerging infectious disease threats. Mendel Douglas Bennett’s Prince Pact Infect Dis. 2015:158.

[19] Hanes D. Using best practices in a clinical guideline for sickle cell anemia. Clin Nurse Spec.2010;24:104.

[20] Ochocinski D Dalal M Black LV. Life-threatening infectious complications in sickle cell disease: a concise narrative review. Front Pediatric. 2020;8:38. 10.3389/fped.2020.00038 PMC7044152 32154192

[21] Kahn KL Mendel P Leuschner KJ. The national response for preventing healthcare–associated infections: research and adoption of prevention practices. Med Care. 2014;52:S33–45. 10.1097/MLR.0000000000000084 24430264

[22] Councill T. Medical art therapy with children. Handb Art Ther. 2003;6:207–19.

[23] Cristina ML Spagnolo AM Giribone L. Epidemiology and prevention of healthcare-associated infections in geriatric patients: a narrative review. Int J Environ Res Public Health. 2021;18:5333. 34067797 10.3390/ijerph18105333 PMC8156303

[24] Sobota A Sabharwal V Fonebi G. How we prevent and manage infection in sickle cell disease. Br J Haematol. 2015;170:757–67. 26018640 10.1111/bjh.13526

